# A Bird’s-Eye View of Bovista

**Published:** 1894-12

**Authors:** C. Carleton Smith


### A BIRD'S EYE VIEW OF BOVISTA.

C. Carleton Smith, M. D.

As a large proportion of our patients are women, a careful study of those drugs that more especially have a bearing upon the diseases of the gentler sex will always well repay us, not only in dollars and cents, but also in the consciousness of being able to cure their numerous ills in a mild, gentle, and enduring manner. One of the most useful remedies in this connection, and one worthy of closer study is Bovista. It ought to be one of the first thought of in the treatment of women who are subject to profuse menstrual flow each month, amounting to a hemorrhage, and especially called for in those cases in which this abnormal flow materially decreases while the sufferer is on her feet, but immediately asserts itself in full force as soon as she lies down *at night.* These women, it has been observed, rarely become pregnant after marriage until Bovista is administered, which cures this inordinate loss of blood, and as a result pregnancy follows next in order, then perfect health. Creosotum causes, also, profuse uterine hemorrhage, worse from lying down, and better getting up and walking about, though not especially at night, as Bovista does. When comparing these two, Creosote should be studied in the case of those persons who show a strong tendency toward phthisis pulmonalis, and who are annually afflicted with winter-cough of a persistent nature, accompanied with spurting of urine. Sabina has profuse menses, but unlike Bovista has large clots, and these of a conical shape, with pain from back to pubis. I should note just here that Sabina patients, when suffering, are often so nervous that they become almost distracted by noises, such for instance as piano-playing. Now sometimes we will meet with cases of menorrhagia, accompanied with large clots of blood looking like pieces of freshly-cut liver, in which the flow invariably becomes fearfully profuse during the night. And allowing ourselves to be guided by this night-aggravation, we might be led to prescribe Bovista, notwithstanding the clots. But study first in such instances Ustilago-maidis, it will probably be the simillimum. Especially useful will it be found in cases of fat, flabby women, who bloat in the face, and who, from repeated losses of blood, show strong dropsical symptoms. A very characteristic guide in the selection of Bovista is a strong odor of onions proceeding from the axillæ, which is quite offensive to the olfactories. Mentally the Bovista patient shows marked absent-mindedness; difficulty also in fixing her attention long on anything; she constantly drops things from her hands; is very desponding and sad; while you talk with her she stares into vacancy, so that you cannot tell whether she is giving any attention to you or not; suffers at intervals with vertigo, which renders her momentarily unconscious; has attacks of nose-bleed from the slightest provocation, such as blowing the nose and sneezing; her face changes color frequently, first very red, then suddenly very pale; very sensitive to drafts of cool air; also worse from cold food.

If at menstrual period the Bovista patient suffers with colic she will bend over, forward, like the Colocynth sufferer. But before you prescribe on that one indication examine the urine first. If it is quite red, and pains are ameliorated by partaking of food, colic is contra-indicated. As I have alluded to Ustilago, I deem it advisable to note here an important symptom peculiar to this drug, and that is, every blunt instrument which the patient uses or handles, such as scissors or pen-knife, leaves a deep indentation in the flesh. This evidently points to a dropsical tendency. And it is in just such women Ustilago acts curatively. Bovista may be compared profitably with the following remedies: Calcarea-carb., Rhus-tox., and Sepia, all of which follow well after its use. Dr. Hering states that the drug in question is very valuable in poisoning from carbon vapors.

